# Management of Long COVID—The CoviMouv' Pilot Study: Importance of Adapted Physical Activity for Prolonged Symptoms Following SARS-CoV2 Infection

**DOI:** 10.3389/fspor.2022.877188

**Published:** 2022-07-01

**Authors:** Claire Colas, Manon Bayle, Pierre Labeix, Elisabeth Botelho-Nevers, Amandine Gagneux-Brunon, Céline Cazorla, Flora Schein, Emma Breugnon, Arnauld Garcin, Léonard Feasson, Frédéric Roche, David Hupin

**Affiliations:** ^1^INSERM, U1059, SAINBIOSE, DVH, Lyon University, Jean Monnet University, Saint-Étienne, France; ^2^Department of Clinical and Exercise Physiology, University Hospital Center, Saint-Étienne, France; ^3^Chaire Santé des Ainés, Presage Institute, Jean Monnet University, Lyon University, Saint-Étienne, France; ^4^Chaire ActiFS, Presage Institute, Jean Monnet University, Lyon University, Saint-Étienne, France; ^5^Department of Infectious Diseases, University Hospital Center, Saint-Étienne, France; ^6^Chaire PreVacCI, Presage Institute, Lyon University, Jean Monnet University, Saint-Étienne, France; ^7^CIRI, Team GIMAP, Univ Lyon, Université Jean Monnet, INSERM, U1111, CNRS, UMR530, Saint-Étienne, France; ^8^Innovation and Pharmacology Clinical Research Unit, University Hospital of Saint-Etienne, Saint-Étienne, France; ^9^Interuniversity Laboratory of Human Movement Biology, EA 7424, Lyon University, Jean Monnet University, Saint-Étienne, France; ^10^Department of Medicine, Solna, Karolinska Institute, Stockholm, Sweden

**Keywords:** COVID-19, long COVID, ongoing symptomatic COVID-19, fatigue, exercise, education, physical capacities

## Abstract

**Context:**

After a COVID-19 infection, some patients have persistent symptoms, the most common is fatigue. To prevent it from becoming chronic (post-COVID-19 syndrome), early management before 3 months could be useful. Exercise and education are recommended.

**Objective:**

To assess fatigue in patients with prolonged symptoms after COVID-19 infection and who received a mixed program of remote adapted physical activity and therapeutic education. The secondary objective was to evaluate the efficacy and safety of this training method thanks to aerobic and anaerobic parameters.

**Methods:**

“CoviMouv': From Coaching in Visual to Mouv in real” is a nonrandomized controlled pilot study. Patients in telerehabilitation followed 12 remote exercise sessions and 3 therapeutic education workshops. Patients on traditional rehabilitation followed their program with a community-based physiotherapist.

**Results:**

Fatigue was reduced after the one-month intervention in both groups (*p* = 0.010). The majority of aerobic parameters were significantly improved, e.g., maximal oxygen uptake (*p* = 0.005), walking distance (*p* = 0.019) or hyperventilation values (*p* = 0.035). The anaerobic parameter was not improved (*p* = 0.400). No adverse event was declared.

**Discussion:**

Telerehabilitation is a good alternative when a face-to-face program is not possible. This care at an early stage of the disease could help prevent the chronicity of post-COVID-19 symptoms and the installation of vicious circles of physical deconditioning. A larger study would be necessary.

## Introduction

Long COVID is a term commonly used to describe “signs and symptoms that continue or develop after acute COVID-19” (NICE Guideline, 2020a). We distinguish two stages in long COVID: (i) ongoing symptomatic COVID-19 for symptoms from 4 to 12 weeks; and (ii) post-COVID-19 syndrome for symptoms that continue for more than 12 weeks and not explained by another diagnosis (NICE Guideline, 2020a). These two forms of long COVID are characterized by multiple symptoms affecting respiratory and cardiovascular capacities, general symptoms such as fatigue, fever and pain, and several other symptoms (e.g., neurological, gastrointestinal, musculoskeletal, and psychological). On the whole, a list of more than 200 symptoms is mentioned, of which persistent fatigue has been found in 39–77% of patients previously infected by the virus (Davis et al., 2020; Townsend et al., 2020).

Post-COVID-19 syndrome can persist beyond 6 months: 76% of hospitalized patients for COVID-19 reporting at least one persistent symptom, and the most common is fatigue or muscle weakness for 63% (Huang et al., 2021). At 12 months, it is always the fatigue that mostly persists (53.1%) with reduced exercise capacity, dyspnoea, concentration problems, and sleeping problems (56.3, 37.5, 39.6, and 26%, respectively; Seeßle et al., 2021). To prevent this post-COVID-19 syndrome and to avoid the chronicity of fatigue, it seems necessary to propose an early intervention during the ongoing symptomatic COVID-19 phase (Townsend et al., 2020; O'Sullivan et al., 2021). There is currently no specific treatment for “acute” asthenia that has progressed for <4 months. However, a program combining exercise and therapeutic patient education has already shown significant benefits for combatting recent or semi recent fatigue following a cardiovascular event (Staniute et al., 2014; Van Geffen et al., 2015; Ter Hoeve et al., 2019) and even during cancer treatment (Van Vulpen et al., 2016; Zhang et al., 2018; Gheyasi et al., 2019). Exercise is also strongly recommended in chronic fatigue syndrome (Clark and White, 2005) and in fibromyalgia, where exercise is the only “strong for” therapy-based recommendation (Macfarlane et al., 2017).

While the resumption of physical activity after COVID-19 infection was controversial at the beginning of the epidemic (NICE Guideline, 2020b), the latest worldwide recommendations for returning to exercise are now unanimous even for patients suffering from long COVID (World Health Organization, 2019; NICE Guideline, 2020a; Haute Autorité de Santé, 2021a; NIHR Themed Review, 2021; World Physiotherapy, 2021). Exercise rehabilitation needs to start earlier and to be adapted to the symptoms with a focus on returning to daily activities and conserving energy (Alberta Health Services, 2019; Faghy et al., 2021). The addition of educational sessions could be beneficial to reduce anxiety (O'Sullivan et al., 2021) and to better manage and monitor the symptoms that may appear during exercise (NICE Guideline, 2020a). To set up this care, a hybrid rehabilitation is recommended with the use of in-home telehealth when the patient has the physical, technical and material capacities (Chartered Society of Physiotherapy, 2021; Ding et al., 2021).

Accordingly, the purpose of this pilot study was to assess fatigue in patients with prolonged symptoms after COVID-19 infection, i.e., during the ongoing symptomatic COVID-19 phase, and who received a mixed program of remote adapted physical activity and therapeutic education. The secondary objective was to evaluate the efficacy and safety of this training method thanks to aerobic and anaerobic parameters.

## Materials and Methods

“CoviMouv': From Coaching in Visual to Mouv in real” is a nonrandomized controlled pilot study carried out on a small scale to evaluate efficiency of the telerehabilitation and its possible adverse effects, in order to determine the sample size of the definitive study. CoviMouv' received approval from the Local Ethics Board (IRBN142021/CHUSTE) and is registered at ClinicalTrials.gov (NCT05236478). Patients were recruited from March to July 2021 and signed a written informed consent. All participants had to be >18 years old, had been hospitalized in a COVID-19 unit at the Saint-Etienne Hospital without being intubated (if hospitalization in intensive care) and had to show post-COVID-19 fatigue for <3 months (COVID-19 infection confirmed by RT-PCR). The exclusion criteria were patients hospitalized in intensive care >72 h, intubated and/or ventilated in intensive care, and patients who were not hospitalized for COVID-19 initially.

### Intervention

**Figure 1:**
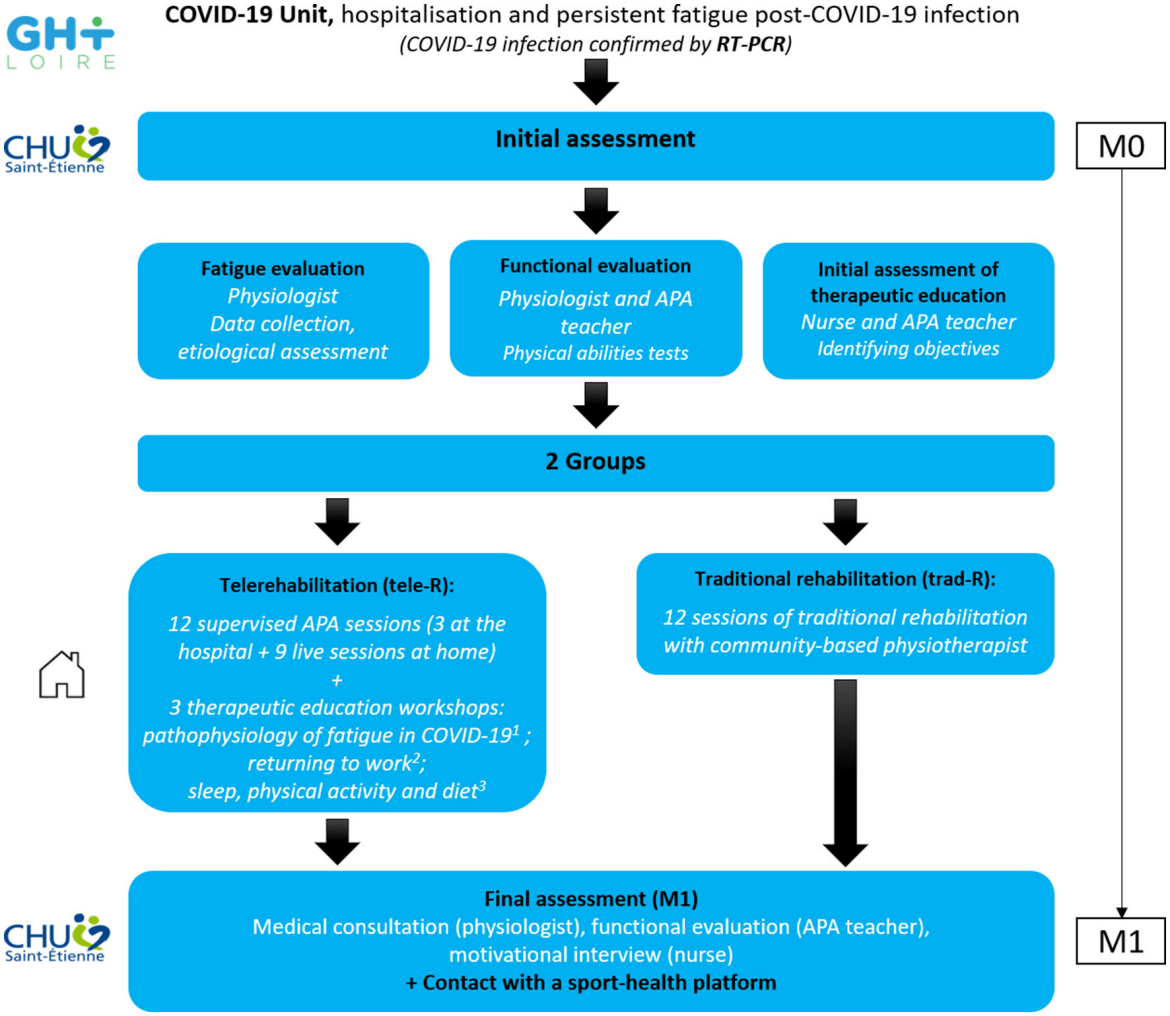
Experimental design of the CoviMouv' study. Pathway of the patients involved in the protocol. ^1^Performed by Internal Medicine or Infections Department. ^2^Performed by Occupational Health Department. ^3^Performed by Physiology Department. RT-PCR, reverse transcription polymerase chain reaction; APA, adapted physical activity.

**Figure 2:**
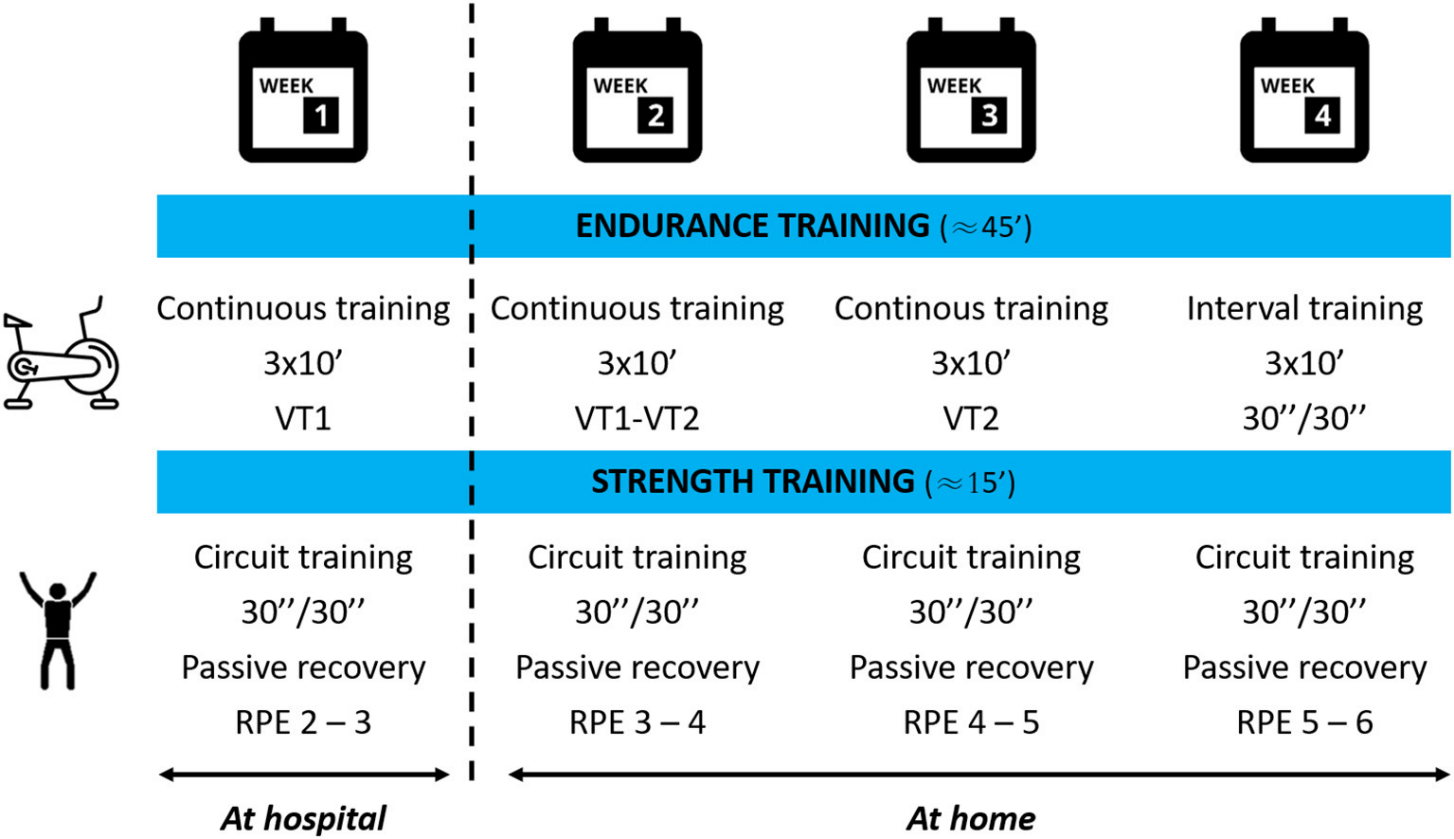
Rehabilitation program of the CoviMouv' study. Aerobic and resistance modalities of a 4-week training program. VT1, ventilatory threshold 1; VT2, ventilatory threshold 2; RPE, rating of perceived exertion, Borg modified scale. X”/X” corresponds to working time/recovery time.

**Figure 3:**
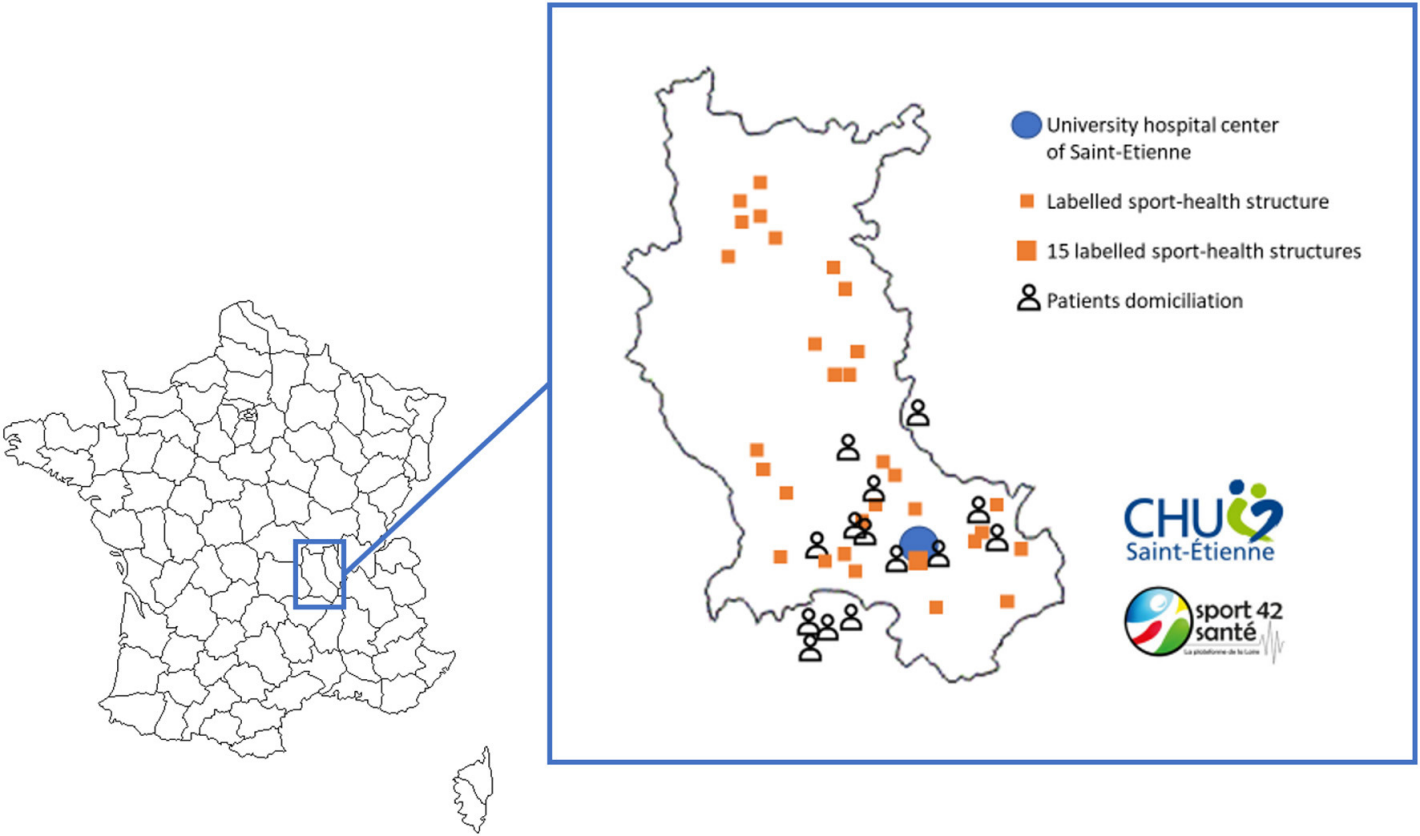
Mapping of Health Sport House (Loire department) and patients' domiciliation. After the CoviMouv' program, patients are invited to sustain an adapted practice of physical activity near their home in labeled structures.

Patients showing post-COVID-19 fatigue have benefitted from a telerehabilitation program (tele-R) at home with 12 supervised personalized exercise sessions and three therapeutic education workshops (Figure 1). Physical rehabilitation was realized at the hospital center the 1st week with an adapted physical activity teacher (three sessions of 1 h, 45 min of aerobic exercise, and 15 min of resistance exercise); then supervised sessions were realized at home by videoconferencing for 3 weeks (three live sessions of 1 h/week, 45 min of aerobic exercise, and 15 min of resistance exercise; Ghram et al., 2022). Aerobic training was performed at ventilatory threshold 1 (continuous training) the 1st week and progressed to intermittent work at ventilatory threshold 2 the last week (Figure 2). Resistance training was performed in whole body circuit training with body weight from a light intensity to a moderate intensity according to perceived exertion using a modified Borg scale from 0 (no exertion) to 10 (extremely hard exertion). The exercise program was personalized based on the results of the functional tests carried out during the initial evaluation. To ensure safety and adapt the intervention for each participant, they wore a connected watch (LifePlus^®^ SAS, Paris, France) to monitor heart rate. Exercise was stopped if the heart rate was >80% maximum heart rate (vigorous exercise) and/or if the perceived exertion >6/10 on modified Borg scale (difficult exercise with breathlessness). The medical follow-up was carried out remotely with a weekly teleconsultation. Psychological and/or dietetic follow-up were proposed during the program, if necessary (weekly remote follow-up). At the end of the program, each participant was invited to contact a sport-health platform in order to continue an adapted physical activity practice near home (Figure 3).

### Materials

**Figure 4:**
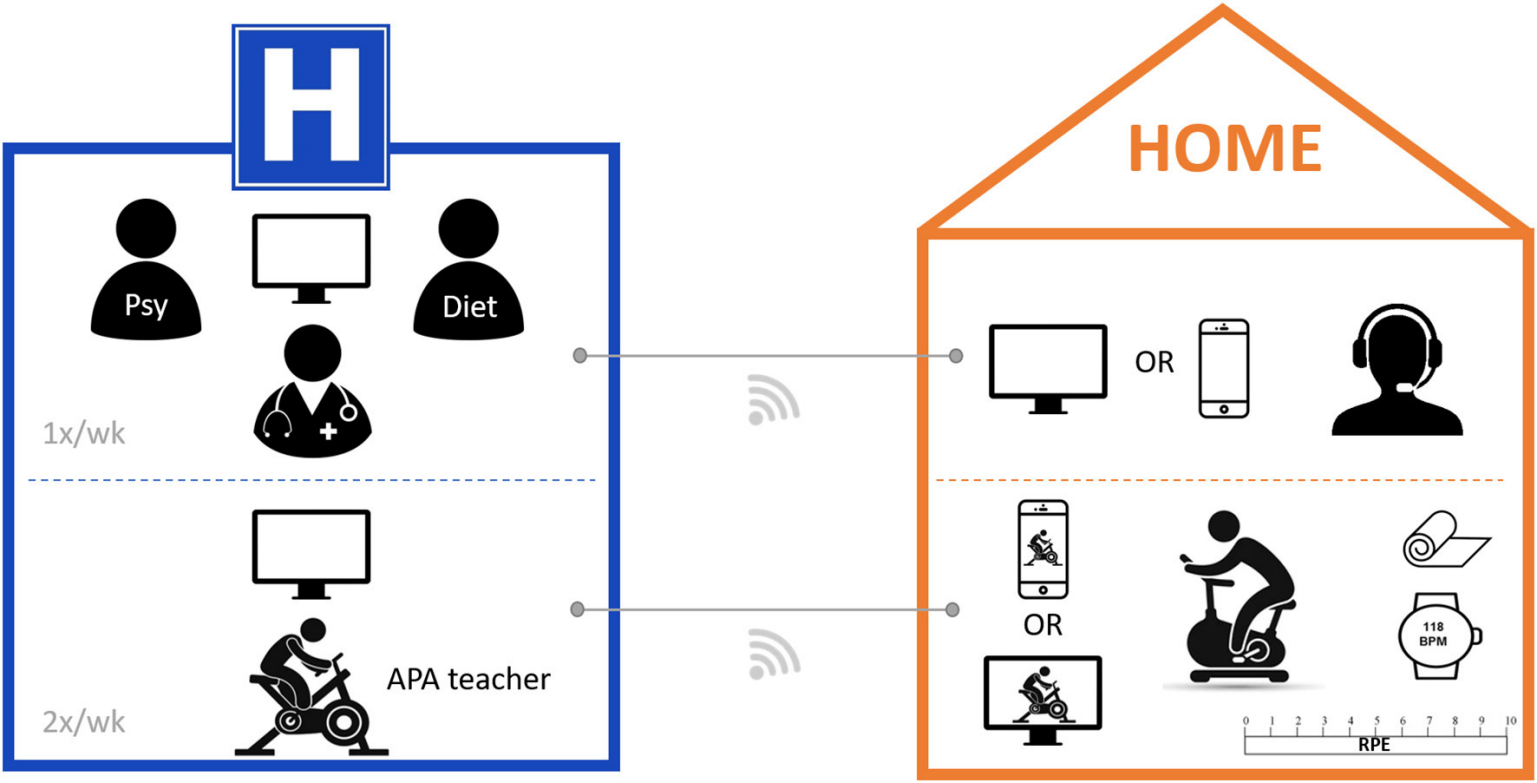
Required equipment for telerehabilitation program of the CoviMouv' study. Material used for remote protocol. Psy, psychologist; Diet, dietitian; APA, adapted physical activity; BPM, beats per minute; RPE, rating of perceived exertion, Borg modified scale.

For the patient, the equipment was composed of a connected device with frontal camera (smartphone, tablet, laptop computer, or computer and webcam) and a microphone (a headset can be used for a better sound quality); a good quality internet connection. For exercise sessions, the patient needed to have an exercise bike, a floor mat, and a sufficient and calm work space. Participants who did not have this equipment at home had to purchase or rent it. The monitoring was realized with a rated perceived exertion scale and a LifePlus^®^ smartwatch lent by the hospital center for the duration of the program. For the professionals, the equipment was composed of a computer or laptop computer with frontal camera; a good quality internet connection; and a secure video conferencing software (Cisco Webex Meetings, Cisco Systems, Inc, San Jose, USA). Regarding privacy, a unique link was sent 48 h before each session by the professional. Access to the meeting was only possible for invited participants. For exercise sessions, the professional needed a cycle ergometer, a floor mat, and a sufficient and calm workspace. Instructions (breathing, change of intensity, and postures) were given during all the sessions and the different parameters (e.g., heart rate, perception of effort, pains, and fatigue) were reported on individual tracking sheets. Materials are detailed in Figure 4.

### Participant Selection

Patients who did not have appropriate equipment and/or internet connection to participate in the program by videoconferencing followed a traditional physical rehabilitation with a community-based physiotherapist that was adapted to symptoms (Haute Autorité de Santé, 2021b). These patients were in the control group. This traditional rehabilitation (trad-R) consisted of three physiotherapy sessions per week for 4 weeks. Content of sessions was adapted to the initial capacities of the patient (delivery of a training booklet with tests results of the initial evaluation) and to his progress over weeks by the physiotherapist.

### Outcome Measures

Assessment of outcomes was undertaken at baseline (M0) and at 1 month (M1) at the end of intervention.

The primary outcome was fatigue evolution assessed using the Chalder Fatigue Score (CFS-11, score between 0 and 33) as recommended by the French High Authority of Health (Haute Autorité de Santé, 2021a). The questionnaire is available in Table 1.

**Table 1:** Chalder fatigue scale (Chalder et al., 1993).

	**Less than usual**	**No more than usual**	**More than usual**	**Much more than usual**
Do you have problems with tiredness?				
Do you need to rest more?				
Do you feel sleepy or drowsy?				
Do you have problems starting things?				
Do you lack energy?				
Do you have less strength in your muscles?				
Do you feel weak?				
Do you have difficulties concentrating?				
Do you make slips of the tongue when speaking?				
Do you find it more difficult to find the right word?				
How is your memory?				

Secondary outcomes were used in order to assess the efficacy of remote training and concerned the following:

- Aerobic performances: Improvement in maximal oxygen uptake (VO_2_ max), maximal aerobic power (MAP) and power at the first ventilatory threshold (VT1) assessed by a cardiopulmonary exercise test (Vyntus CPX, CareFusion, San Diego, California, USA) from baseline to completion of the one-month intervention; forced expiratory volume in 1 s (FEV-1) assessed by spirometry; walking distance at 6-min walk test (6-MWT, in m). Values of ventilation per unit of carbon dioxide production slope (VE/VCO_2_) and end-tidal carbon dioxide pressure at rest (PetCO_2_ rest), at peak (PetCO_2_ peak) and at recovery (PetCO_2_ recovery) were measured in order to assess the presence of hyperventilation syndrome. A VE/VCO_2_ slope below 29 suggested a good prognosis and under 45 a bad prognosis. PetCO_2_ values below 35 mmHg were indicative of hyperventilation. We also calculated the change in PetCO_2_ from rest to peak (ΔPetCO_2_ rest/peak) and from peak to recovery (ΔPetCO_2_ peak/recovery).
- Anaerobic performances: Change in muscular strength was assessed by a handgrip test (muscular strength of biceps in kg) with a Jamar hydraulic hand dynamometer (JLW instruments, Chicago, USA).

### Data Analysis

Statistical analysis was performed using Jamovi statistical software (version 1.1.9.0). Data were checked for normality and homogeneity of variances using Shapiro-Wilk and Levene tests, respectively. According to the Shapiro-Wilk test, a two-way repeated measures ANOVA or a Friedman test was performed. Where a significant interaction difference occurred, Tukey's *post hoc* analyses were performed. The level of significance was set at *p* < 0.05.

## Results

**Figure 5:**
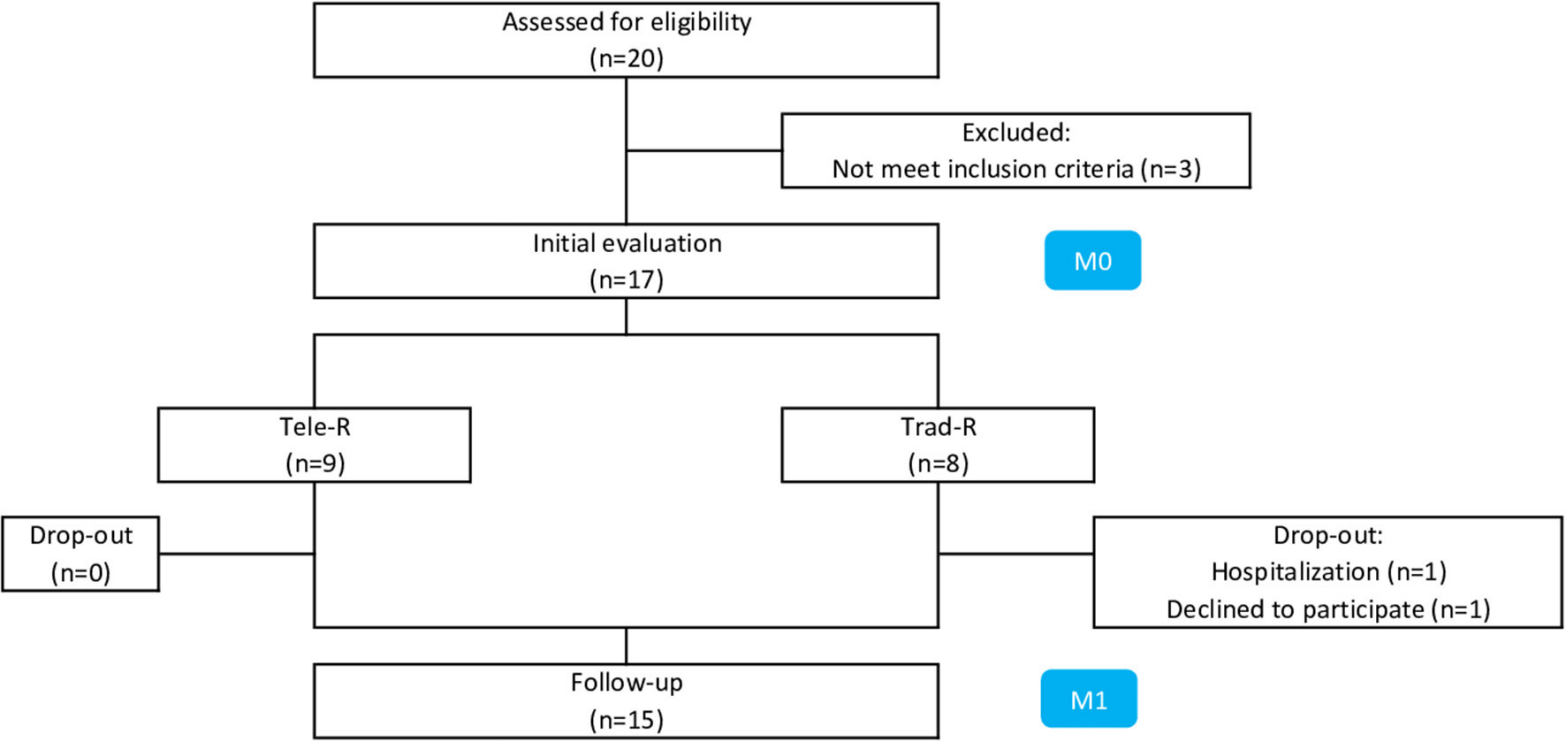
Flowchart. Tele-R, telerehabilitation group; Trad-R, traditional rehabilitation group.

In total, 17 patients (nine men and eight women) were recruited and assigned to tele-R (*n* = 9) or trad-R (*n* = 8) groups. Two trad-R patients did not return for post-testing. Thus, data were collected on nine subjects in the tele-R group and six subjects in the trad-R group (Figure 5). Participants achieved an average of 81 ± 9% and 78 ± 25% of the sessions for tele-R and trad-R groups, respectively. There were no significant differences between both groups at baseline (Table 2). No adverse events were declared.

**Table 2:** Characteristics of the study population.

	**Tele-R (*n* = 9)**	**Trad-R (*n* = 6)**	
**Characteristics (unit)**	**Mean (SD)**	**Mean (SD)**	***p*** **-value**
Age (y)	52.2 (12.8)	52.0 (12.3)	0.971
Height (m)	169 (7.88)	175 (8.92)	0.164
Weight (kg)	72.2 (19.4)	87.0 (11.0)	0.116
BMI (kg.m^−2^)	25.1 (4.98)	28.8 (6.08)	0.220
CFS-11	25.2 (5.26)	27.6 (4.16)	0.403
VO_2_ (mlO_2_.kg^−1^.min^−1^)	21.7 (4.91)	23.2 (7.63)	0.637
MAP (W)	111 (22.0)	140 (40.0)	0.235^a^
VT1 (W)	51.1 (16.2)	70.0 (19.1)	0.050
6-MWT (m)	463 (112)	431 (111)	0.576
Handgrip (kg)	30.8 (13.3)	36.4 (13.0)	0.459
FEV-1 (%)	91.8 (13.5)	86.5 (11.4)	0.445
Hospitalization duration (d)	8.9 (4.0)	7.8 (4.9)	0.678^a^

*Tele-R, telerehabilitation group; Trad-R, traditional rehabilitation group; BMI, body mass index; CFS-11, Chalder Fatigue Score; VO_2_, maximal oxygen uptake; MAP, maximal aerobic power; VT1, power at the first ventilatory threshold; 6-MWT, 6-min walk test; FEV-1, forced expiratory volume in 1 s*.

a *Mann-Whitney test was used for MAP and hospitalization duration data because of the non-equal variance and non-normality, respectively*.

The mean age of our sample was 52.1 ± 12.2 years old. Regarding COVID-19 infection, the average time of hospitalization was 8.1 ± 4.4 d.

### Fatigue

CFS-11 was reduced after the one-month intervention in both tele-R (25.2 ± 5.3 vs. 21.1 ± 5.1) and trad-R (27.6 ± 4.2 vs. 26.2 ± 5.2) groups, showing a significant time effect (*p* = 0.010) without a difference between groups.

### Aerobic Performances

At M0, 11/15 patients showed reduced exercise capacity with low peak VO_2_ [≤ 85% of predicted value (Barbara et al., 2022)] vs. 4/15 at M1. One patient even had severe limitations with a predicted VO_2_ value <60% at M0, which improved at M1. An improvement of VO_2_max of 3.8 points (+18 ± 15%) and 4.1 points (+17 ± 19%) was observed in tele-R and trad-R, respectively. The ANOVA showed a significant time effect (*p* = 0.005). However, there was no group effect and no time^*^group interaction.

**Figure 6:**
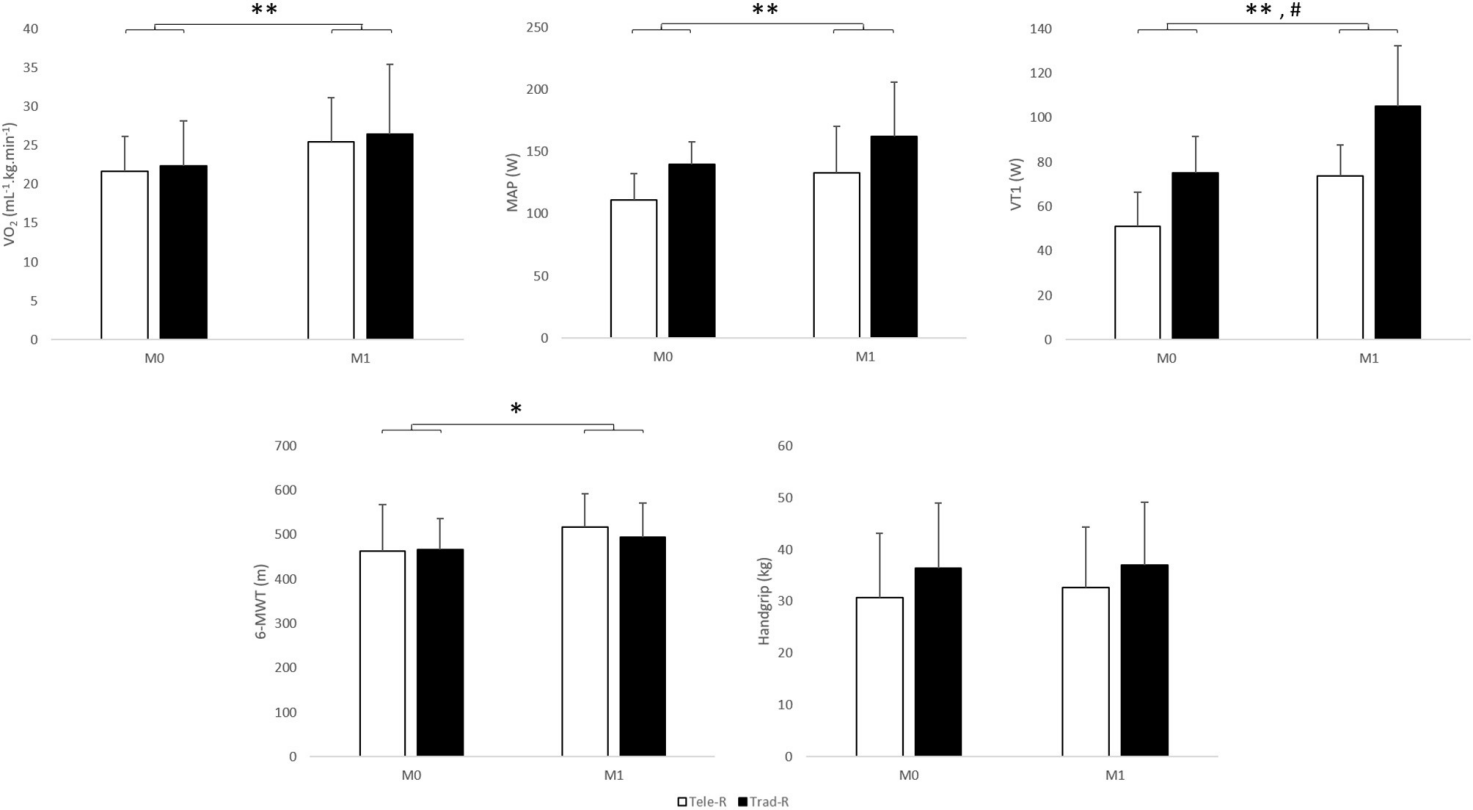
Aerobic and anaerobic physiological variables before and after a one-month exercise program. The ANOVA showed a significant time effect for all variables and a group effect for VT1 only. No significant interaction time*group was found. Tele-R, telerehabilitation group; Trad-R, traditional rehabilitation group; VO_2_, maximal oxygen uptake; MAP, maximal aerobic power; VT1, ventilatory threshold 1; 6-MWT, 6-min walk test. *Time effect (*p* < 0.05). **Time effect (*p* < 0.01). ^#^Group effect (*p* < 0.01).

There was also a significant time effect for all aerobic performance data, i.e., MAP (22 ± 17 vs. 17 ± 18%, *p* = 0.004), VT1 (60 ± 71 vs. 41 ± 33%, *p* = 0.001), FEV-1 (14 ± 12 vs. 6 ± 7%, *p* = 0.020), and 6-MWT (15 ± 15 vs. 6 ± 4%, *p* = 0.019). There was also a group effect for VT1 (*p* = 0.006, η^2^*p* = 0.454) but no time^*^group interaction (Figure 6). ΔVO_2_, ΔMAP, ΔVT1, and Δ6-WMT were not significantly different between groups.

Regarding hyperventilation values, VE/VCO_2_ were under 29 for 2/15 patients (i.e., without hyperventilation signs), 12/15 were between 29 and 45, and 1/15 was >45 (i.e., with bad prognosis). At M1, VE/VCO_2_ decreased by 3 ± 18%; the ANOVA found no significant difference in relation to a strong disparity of values. However, 2/15 patients were still under 29 and none was above 45 at M1. Regarding PetCO_2_ values, all of our sample showed signs of hyperventilation at rest, and 14/15 patients at peak and at recovery during M0 cardiopulmonary exercise test. At M1, all still had PetCO_2_ rest values <32, and 13/15 patients still had low values at peak and at recovery. There was a time effect for the majority of parameters, i.e., PetCO_2_ rest (*p* = 0.035), PetCO_2_ peak (*p* = 0.020), PetCO_2_ recovery (*p* = 0.014), and ΔPetCO_2_ peak/recovery (*p* < 0.009) increased significantly from M0 to M1. ΔPetCO_2_ rest/peak was not modified over time (*p* = 0.055). Moreover, PetCO_2_ rest showed a time^*^group interaction (*p* = 0.024) revealing a significant increase between M0 and M1 for the trad-R group with Tukey's *post-hoc* test (*p* = 0.033).

### Anaerobic Performance

Muscular strength assessed by handgrip was not improved in both groups (9 ± 16 vs. 2 ± 16%, *p* = 0.400). Δhandgrip was not significantly different between groups (Figure 6).

## Discussion

The objective of our study was to assess fatigue, the most persistent symptom in the different forms of long COVID, after a mixed program of telerehabilitation and therapeutic education for patients with ongoing symptomatic COVID-19, i.e., with persistent symptoms of <3 months following a COVID-19 infection. Our results showed an improvement in CFS-11 in both tele-R and trad-R groups. Managing fatigue through exercise could be an effective solution for these patients with persistent symptoms. To our knowledge, this is the first study which has assessed the benefits of adapted physical activity for persistent symptoms of COVID-19, particularly for fatigue. Barbara et al. (2022) recently demonstrated the benefit of a combination of aerobic and resistance training on both cardiorespiratory and musculoskeletal fitness in long COVID-19 patients “with several months lasting symptoms.” After 8 weeks of exercise training, VO_2peak_ and muscle strength increased (Barbara et al., 2022). Thus management of long COVID-19, even away from infection, could be beneficial for health. In the literature, we found two case reports about the effects of exercise showing an improvement in physical function, muscle strength and exercise capacity, but no benefits on fatigue or quality of life for one (Mayer et al., 2021), and an improvement in cardiopulmonary function, muscle strength and fatigue for the other (Longobardi et al., 2022).

Regarding the efficacy and security of our remote program, this pilot study has demonstrated benefits of telerehabilitation on several parameters of physical capacities such as VO_2_max, maximal aerobic power or walking distance. Anaerobic capacities were not enhanced. These parameters were increased in both groups, showing that personalized exercise is effective even in this original remote training method if supervised by a professional. Our results are comparable to other studies that showed equal benefits of home-based telerehabilitation in comparison with center-based rehabilitation (Maddison et al., 2019; Batalik et al., 2020; Fanget et al., 2022). Furthermore, people with prolonged post-COVID symptoms have often demonstrated reduced exercise capacity (VO_2_ values ≤ 85% of predicted values). This was the case for 73% of our sample, which is consistent with the 75% of a larger sample assessed in the study by Motiejunaite et al. (2021). This exercise impairment was reversed for 64% of our subjects after 1 month of personalized exercise. Thus a holistic telerehabilitation program offering adapted physical activity and education, supervised by a team of experts, seems to be a good alternative rehabilitation program when a face-to-face program is not possible, and without adverse events detected in our sample.

Special attention was paid to the hyperventilation values. All of our patients had high values of ventilation per unit of carbon dioxide production and/or low values of resting end-tidal carbon dioxide pressure. This feature was also observed at peak exercise for 24% of patients by Motiejunaite et al. (2021). Wood et al. (2021) also reported low levels of end-tidal carbon dioxide despite normal respiratory rate in individuals with long COVID. In our study, a one-month rehabilitation program at hospital or in community-based center showed an improvement in the majority of parameters of hyperventilation syndrome; which was also found in patients with persistent symptoms after a COVID-19 infection and who underwent a respiratory rehabilitation program (Bouteleux et al., 2021). However, we note nevertheless that the values remained below the thresholds of good prognosis in our sample.

The main strength of our study was the originality of the program, offering a hybrid rehabilitation with adapted physical activity and education combined with face-to-face and remote interventions. This care at an early stage of the disease could help to prevent the chronicity of post-COVID-19 symptoms and the installation of vicious cycles of physical deconditioning, which itself promotes fatigue. This integrative and person-centered approach is also the one recently recommended by Roth et al. (2021).

Our pilot study presents limitations and the first is the reduced size of our sample. We have been limited by the end of the third wave of infection in France and a decrease in the number of patients previously hospitalized and in the ongoing symptomatic COVID-19 phase. However, these first results showed that an early intervention can improve physical function and reduce fatigue for patients with persistent symptoms after a COVID-19 infection. However, we lack a control group without rehabilitation to compare changes over time and evaluate the interest of this early management for patients. In addition, there was no randomization for tele-R and trad-R (the allocation was made arbitrarily based on a patient's sports and computer equipments), and no differences were found at baseline between two groups. This non-randomization may raise issues of socioeconomic differences for those who do not have access to the internet or who have limited computer skills, for example. Finally, it is important to highlight the debate concerning the existence or not of a long COVID. A recent study questions the attribution of persistent symptoms to a COVID-19 infection (Matta et al., 2022). In our study, we limited this bias by including patients referred by COVID-19 services of the hospital and whose COVID-19 infection had been confirmed by RT-PCR. Moreover, the existence of recommendations from major entities such as the World Health Organization, the National Institute for Health and Care Excellence, and the French High Authority of Health underline the importance of recognizing and managing symptoms to avoid anxiety and medical errancy. The association of adapted physical activity and education would allow a better understanding and control of persistent symptoms.

Thus, a larger study would be necessary to assess the relevance of exercise training in telerehabilitation of patients with persistent symptoms after COVID or long COVID. Furthermore, longer term follow-up would be necessary in order to assess if the benefits of this telerehabilitation persist over time. This is the subject of our next randomized study.

## Data Availability Statement

The raw data supporting the conclusions of this article will be made available by the authors, without undue reservation.

## Ethics Statement

The studies involving human participants were reviewed and approved by Comité d'Ethique du CHU de Saint-Etienne—Commission Recherche de Terre d'Éthique Ref: IRBN142021/CHUSTE. The patients/participants provided their written informed consent to participate in this study.

## Author Contributions

CCo, PL, MB, and DH contributed to the acquisition of data. CCo wrote the first draft of the manuscript. DH provided critical revision for intellectual content and oversight. All authors reviewed and approved the final version of the manuscript.

## Conflict of Interest

The authors declare that the research was conducted in the absence of any commercial or financial relationships that could be construed as a potential conflict of interest.

## Publisher's Note

All claims expressed in this article are solely those of the authors and do not necessarily represent those of their affiliated organizations, or those of the publisher, the editors and the reviewers. Any product that may be evaluated in this article, or claim that may be made by its manufacturer, is not guaranteed or endorsed by the publisher.
